# Neurological symptoms and axonal damage in COVID-19 survivors: are there sequelae?

**DOI:** 10.1007/s12026-021-09220-5

**Published:** 2021-08-07

**Authors:** Silvia Bozzetti, Sergio Ferrari, Serena Zanzoni, Daniela Alberti, Michele Braggio, Sara Carta, Francesco Piraino, Daniele Gabbiani, Domenico Girelli, Riccardo Nocini, Salvatore Monaco, Ernesto Crisafulli, Sara Mariotto

**Affiliations:** 1 Neurology Unit, Department of Neurosciences, Biomedicine and Movement Sciences, University of Verona, Policlinico GB Rossi, P.le LA Scuro 10, 37134 Verona, Italy; 2 Centro Piattaforme Tecnologiche, University of Verona, Verona, Italy; 3 School of Medicine in Sports and Exercise, University of Verona, Verona, Italy; 4 Quanterix Corporation, Billerica, MA 01821 USA; 5 Department of Medicine, Section of Internal Medicine, University of Verona and Azienda Ospedaliera Universitaria Integrata of Verona, Verona, Italy; 6 Department of Otolaryngology-Head and Neck Surgery, University Hospital of Verona, Verona, Italy; 7 Department of Medicine, Respiratory Medicine Unit, University of Verona and Azienda Ospedaliera Universitaria Integrata of Verona, Verona, Italy

**Keywords:** Neurofilament, COVID-19, NfL, Hyposmia, Hypogeusia, SARS-CoV-2

## Abstract

The persistence of neurological symptoms after SARS-CoV-2 infection, as well as the presence of late axonal damage, is still unknown. We performed extensive systemic and neurological follow-up evaluations in 107 out of 193 consecutive patients admitted to the COVID-19 medical unit, University Hospital of Verona, Italy between March and June 2020. We analysed serum neurofilament light chain (NfL) levels in all cases including a subgroup (*n* = 29) of patients with available onset samples. Comparisons between clinical and biomarker data were then performed. Neurological symptoms were still present in a significant number (*n* = 49) of patients over the follow-up. The most common reported symptoms were hyposmia (*n* = 11), fatigue (*n* = 28), myalgia (*n* = 14), and impaired memory (*n* = 11) and were more common in cases with severe acute COVID-19. Follow-up serum NfL values (15.2 pg/mL, range 2.4–62.4) were within normal range in all except 5 patients and did not differentiate patients with vs without persistent neurological symptoms. In patients with available onset and follow-up samples, a significant (*p* < 0.001) decrease of NfL levels was observed and was more evident in patients with a severe acute disease. Despite the common persistence of neurological symptoms, COVID-19 survivors do not show active axonal damage, which seems a peculiar feature of acute SARS-CoV-2 infection.

## Introduction

A wide spectrum of neurological symptoms has been described in patients with novel SARS-CoV-2 infection. These include specific syndromes as encephalitis, neurovascular disorders, neuropathies, and muscular diseases as well as non-specific symptoms as headache, vertigo, fatigue, hyposmia, and hypogeusia [1, 2]. These data raised concerns about the possible occurrence of long-term neurological sequelae, supported by the persistence of hyposmia and hypogeusia.

Neurofilament light chain (NfL) levels are sensitive biomarkers of axonal damage, increased in patients with central and peripheral nervous system disorders [3]. An increase of serum or CSF NfL levels has been demonstrated also in subjects with acute SARS-CoV-2 infection, in particular in those with severe COVID-19 syndrome, with central neurological symptoms, and in patients with an unfavourable short-term outcome [4–7]. NfL levels are increased also in cases with mild to moderate COVID-19 and in patients without evidence of neurological symptoms, further confirming the co-occurrence of neuronal damage in this condition [4, 8].

To detect the possible persistence of long-term neurological sequelae, we herein performed a clinical and biomarker follow-up study of patients with antecedent confirmed SARS-COV-2 infection including also an extensive comparison with acute stage evaluations in a subgroup of patients.

## Patients and methods

### Study subjects

Consecutive patients (*n* = 193) with SARS-CoV-2 infection, confirmed by positive reverse transcriptase PCR of nasopharyngeal swab, were admitted to the COVID-19 medical unit of the University Hospital of Verona, Italy between March and June 2020. Among these, 107 patients without concomitant neurological comorbidities (i.e. cognitive impairment, cerebrovascular diseases, peripheral neuropathies) consented to a follow-up evaluation which included physical examination, respiratory tests, questionnaires related to the persistence on neurological specific and non-specific symptoms, and laboratory re-evaluation 6 months after symptoms onset and were therefore included in the present study. In 29 out of 107 included patients, both acute and follow-up clinical and biomarker data were available for comparison analyses.

### Clinical data and testing

Demographic and clinical data were collected in each included case in a standardised form.

Lung function was analysed according to international recommendations. A flow-sensing spirometer connected to a computer (Jaeger MasterScreen PFT System) was used for the measurements. Forced vital capacity (FVC), forced expiratory volume in the first second (FEV_1_), and total lung capacity (TLC) were recorded taking FEV_1_/FVC ratio as index of airflow obstruction. Data concerning arterial blood gas analysis in term of arterial partial oxygen pressure (PaO_2_) and arterial partial carbon dioxide pressure (PaCO_2_) were also collected. Walking capacity was assessed by the distance covered at the 6-min walking test (6MWD). Individual’s perceived dyspnoea and fatigue at baseline and at end-effort of the 6-min walking test were measured with a 10-point modified Borg scale.

### NfL analysis

Serum NfL levels were analysed in duplicates in all follow-up sera and in available paired onset sera by investigators blinded to clinical data using SIMOA Nf-light® kit in SR-X immunoassay analyser, Simoa™ (Quanterix Corporation, Billerica, MA, USA), as previously described [9]. Intra-assay coefficient of variation was < 8%.

### Statistical analysis

A preliminary Shapiro–Wilk test was performed. Data are reported with numbers (percentages) for categorical variables and mean (SD) or median [interquartile range] for continuous variables with a normal or non-normal distribution, respectively. Categorical variables were compared using the χ^2^ test or the Fisher exact test, while continuous variables were assessed using the independent *t*-test or the non-parametric Mann–Whitney *U* test or Wilcoxon test when appropriate. All analyses were performed using IBM SPSS, version 25.0 (IBM Corp., Armonk, NY, USA) and a *p* value of < 0.05 has been considered statistically significant.

## Results

Among 107 included patients, 70 were males and median age was 63 years (range 32–90). During the acute COVID-19 phase, 16 patients required admission to ICU, mean C-reactive protein was 75.8 mg/L (SD 59.3), and mean P/F ratio was 279.86 (SD 78.5). None of these patients reported specific neurological symptoms suggestive for encephalitis, neurovascular disorders, neuropathies, and muscular diseases, while 37 subjects complained of hyposmia, 55 of hypogeusia, 11 of vertigo, 47 of fatigue, 20 of headache, 26 of myalgia, 7 of impaired consciousness, 6 of impaired memory, and 10 of syncope. Among patients with available onset and follow-up samples (*n* = 29), 14 cases had increased NfL levels in the acute stage in comparison with a group of healthy controls analysed in our laboratory (*n* = 60) whose NfL values are in agreement with those previously established for age-matched healthy controls [10]. In addition, NfL levels at onset did not show any correlation with gender and age.

On follow-up re-evaluation, 49 patients complained of the persistence of at least one possible neurological symptom (hyposmia, *n* = 11; hypogeusia, *n* = 7; vertigo, *n* = 6; fatigue, *n* = 28; headache, *n* = 4; myalgia, *n* = 14; impaired memory, *n* = 11). Mean follow-up serum NfL value was 15.2 pg/mL (range 2.4–62.4) and only 5 patients had increased NfL values in comparison with healthy controls. None of these 5 patients reported hyposmia or hypogeusia but 3 of them had persistent fatigue.

No significant differences in functional biomarkers of respiratory outcome, age, body mass index (BMI), creatinine, serum C-reactive protein levels, and serum NfL levels were observed in patients with vs without persistent neurological symptoms. Interestingly, patients reporting at least one possible neurologic symptom at follow-up had experienced a significantly more prolonged duration of systemic COVID-19 (49.0 days vs 27.5, *p* < 0.01). A significant correlation (*p* < 0.001) was noted between NfL levels obtained during the follow-up and age in absence of any correlation with gender.

**Fig. 1:**
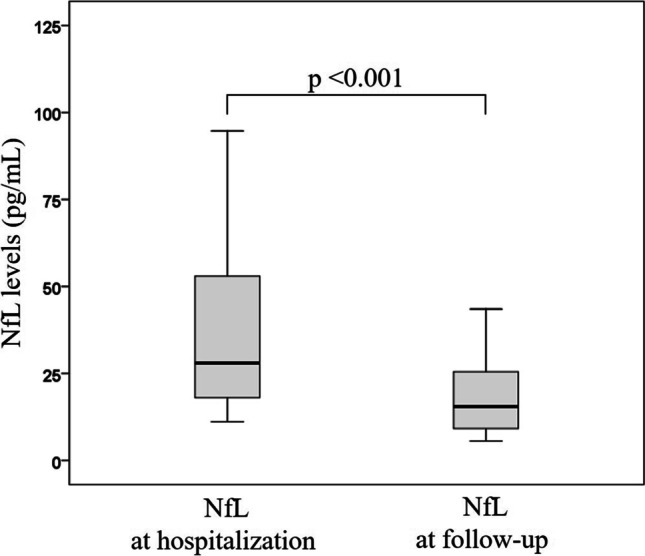
Comparison between NfL levels measured during the acute COVID-19 stage and over the follow-up. A significant decrease of serum NfL values was observed on follow-up measurement in patients with available onset and follow-up samples (*n* = 29), thus suggesting the absence of persistent active axonal damage. The boxes show median values and interquartile range for each group

A significant decrease (*p* < 0.001, mean difference 32.8 pg/mL, standard deviation 56 pg/mL) of NfL values was observed over time in patients with available onset and follow-up sera, as shown in Fig. 1. In particular, in 14 out of 29 patients, a 50% decrease of NfL levels was observed. In this subgroup of patients, a significant difference in terms of previous ICU admission (*p* < 0.001), BMI (*p* = 0.045), Borg scale dyspnoea at end-effort (*p* = 0.003), and duration of hospitalisation (*p* = 0.049) was observed. Notably, none of demographic, anamnestic, respiratory variables, and neurologic symptoms distinguished patients with a 50% decrease of serum NfL levels (Table 1).

**Table 1:** Comparison of serum NfL levels reduction in patients with available onset and follow-up evaluations

Variables	Patients with ≥ 50% reduction of serum NfL levels (***n*** = 14)	Patients with < 50% reduction of serum NfL levels (***n*** = 15)	*p* value
Age, years	62 ± 10.8	70.6 ± 9.3	**0.030**
Sex, male, ***n*** (%)	11 (79)	9 (60)	0.427
BMI, kg∙m^2^	28.7 [6.3]	26. 9 [1.9]	**0.045**
Former smokers, ***n*** (%)	6 (43)	6 (40)	0.876
Arterial hypertension, ***n*** (%)	8 (57)	10 (67)	0.597
Extensional dyspnoea, ***n*** (%)	3 (21)	1 (6.7)	0.330
Hyposmia, ***n*** (%)	1 (8.3)	1 (6.7)	> 0.999
Hypogeusia, ***n*** (%)	1 (8.3)	1 (6.7)	> 0.999
Vertigo, ***n*** (%)	2 (16)	0 (0)	0.188
Fatigue, ***n*** (%)	4 (33)	4 (27)	> 0.999
Myalgia, ***n*** (%)	3 (27)	2 (13)	0.620
Impaired memory, ***n*** (%)	4 (33)	1 (6.7)	0.139
At least one neurologic symptom, ***n*** (%)	7 (58)	5 (33)	0.194
Other neurologic symptoms^§^, ***n*** (%)	4 (33)	3 (20)	0.662
Symptoms duration, days	66 [100]	27 [26]	0.052
Timespan between sampling and symptoms resolution, days	122 [121]	124 [27]	0.462
C-reactive protein, mg/L	2 [4.5]	1 [3]	0.649
FEV_1_, % pred	126.4 ± 21.8	128.1 ± 22	0.835
FVC, % pred	127.5 ± 22.4	134.5 ± 24.1	0.441
FEV_1_/FVC	103.2 ± 8.5	100.2 ± 9	0.371
TLC, % pred	103 ± 11.7	104 ± 14	0.841
DL_CO_, % pred	82.7 ± 16.6	94 ± 21.8	0.140
K_CO_, % pred	88 ± 18.2	97 ± 16.2	0.180
PaO_2_, mmHg	95.2 ± 9.4	93.2 ± 19.6	0.731
PaCO_2_, mmHg	38 [4]	39 [3]	0.642
6MWD, total distance covered	525 [214]	530 [270]	0.163
6MWD, % pred	96.5 ± 16.5	100.2 ± 24.4	0.640
Borg scale dyspnoea at end-effort*, score	4 [3]	1 [2]	**0.003**
Borg scale fatigue at end-effort*, score	4 [4]	1 [2]	0.165
SpO_2_ at baseline, %	97.3 ± 1.4	96.8 ± 1.6	0.458
SpO_2_ at end-effort, %	97 [3]	96 [2]	0.964
Length of hospital stay, days	24 [21]	11 [9]	**0.049**
Previous ICU admission, ***n*** (%)	10 (71)	1 (6.7)	** < 0.001**

Data are shown as mean ± standard deviation, median [interquartile range], or number (percentages). In bold, significant variables

Atrial fibrillation, ischemic heart disease, chronic heart failure, diabetes mellitus, chronic obstructive pulmonary disease, asthma, and chronic kidney disease have a prevalence of < 10% with no significant difference between groups

^§^Other self-reported symptoms included sleep disorders, paresthesia, and unspecific sight deficit

^*^The median of Borg scale at baseline was 0 in both groups

Abbreviations:* BMI*, body mass index; *FEV*_*1*_, forced expiratory volume at 1st second; *FVC*, forced vital capacity; *TLC*, total lung capacity; *DL*_*CO*_, diffusion capacity for carbon monoxide; *K*_*CO*_, carbon monoxide transfer coefficient; *PaO*_*2*_, arterial partial oxygen pressure; *PaCO*_*2*_, arterial partial carbon dioxide pressure; *6MWD*, 6-min walking distance; *SpO*_*2*_, oxygen saturation by pulse oximetry; *ICU*, intensive care unit

## Discussion

To the best of our knowledge, our study is the first which explores the neurological sequelae of SARS-CoV-2 infection from a clinical and biomarker perspective after a consistent follow-up, set at 6 months to avoid the long-term effect of the acute neurological event on NfL values, in accordance with previous studies [11, 12].

Specific and non-specific neurological symptoms have been reported in patients with COVID-19 [1, 2] and different studies documented an increase of NfL levels during the acute phase of the infection, thus supporting the occurrence of concomitant acute axonal damage [4, 5, 7, 8]. In addition, despite the correlation between NfL values and the severity of the clinical picture in the acute stage [4, 6], the potential association between NfL values and long-term neurological sequelae is still unclear.

Our data reveal that only 4.6% of COVID-19 survivors display increased NfL levels over the follow-up, in contrast with the significantly higher percentage (57%) of patients with increased NfL values previously observed in the acute phase and herein confirmed [4]. In addition, NfL levels decrease over the follow-up in the vast majority of cases with available repetitive measurements. Then, a clear NfL/age correlation was noted only during recovery, thus emphasising the significant impact of disease severity on NfL values in the acute phase. Our data are in agreement with previous studies on HIV-infected patients, where a reduction of NfL values to normal range over time, and in particular during antiretroviral therapy, has been described [13].

These observations do not support the persistence of axonal damage in COVID-19 survivors and retain significant clinical implications. Interestingly, despite this finding, almost half of patients herein analysed still complain of at least one non-specific neurological symptom. These self-reported symptoms do not correlate with the persistence of systemic/metabolic symptoms or signs but significantly correlate with the severity of acute SARS-CoV-2 infection, thus potentially reflecting the consequences of the acute event. In accordance, when analysing both onset and follow-up samples, the most significant NfL reduction occurs in patients having experienced a more severe acute disease (i.e. longer hospitalisation or ICU admission). In addition, neither follow-up NfL levels nor their reduction over time correlates with the persistence of non-specific neurological symptoms, thus not supporting a neurological nature of these symptoms. This finding is in agreement with previous studies reporting increased NfL levels during acute COVID-19 also in patients not reporting neurological symptoms, those suggesting the occurrence of a subclinical neuronal damage [4].

## Conclusions

COVID-19 survivors frequently complain of non-specific neurological symptoms but do not present a prolonged axonal damage. A substantial decrease of NfL values in comparison with onset levels is observed in this condition, and in particular in patients with a more severe acute event. Our study has some limitations including the small sample size of patients with available onset and follow-up sera and the inability to obtain brain imaging data and to compare COVID-19-related complications with those observed in other infectious disorders.

However, our data give further cues to clarify the nature of non-specific neurological symptoms and the possible persistence of long-term neurological sequelae.

## Data Availability

The data that support this study are available for sharing and further examinations from the corresponding authors (S.M.) on reasonable requests. The data are not publicly available because they contain information that could compromise patients’ consent.
